# Supplementary material for: Structure and mechanism of potent bifunctional β-lactam- and homoserine lactone-degrading enzymes from marine microorganisms
Source: Sci Rep. 2020 Jul 30;10:12882. doi: 10.1038/s41598-020-68612-z (PMC7392888; doi:10.1038/s41598-020-68612-z)
Supplement: Supplementary file 1 — Supplementary information. [file 41598_2020_68612_MOESM1_ESM.docx]

## Structure and mechanism of potent bifunctional β-lactam- and homoserine lactone-degrading enzymes from marine microorganisms

**Supplemental Data**

Christopher Selleck,^a^ Marcelo Monteiro Pedroso,^a,b,c,†^ Liam Wilson,^a^ Stephan Krco,^a^ Esmée Gianna Knaven,^a^ Manfredi Miraula,^a,d^ Nataša Mitić,^d^ James A. Larrabee,^e^ Thomas Brück,^f^ Alice Clark,^c^ Luke W. Guddat,^a^ Gerhard Schenk^a,b,c,†^


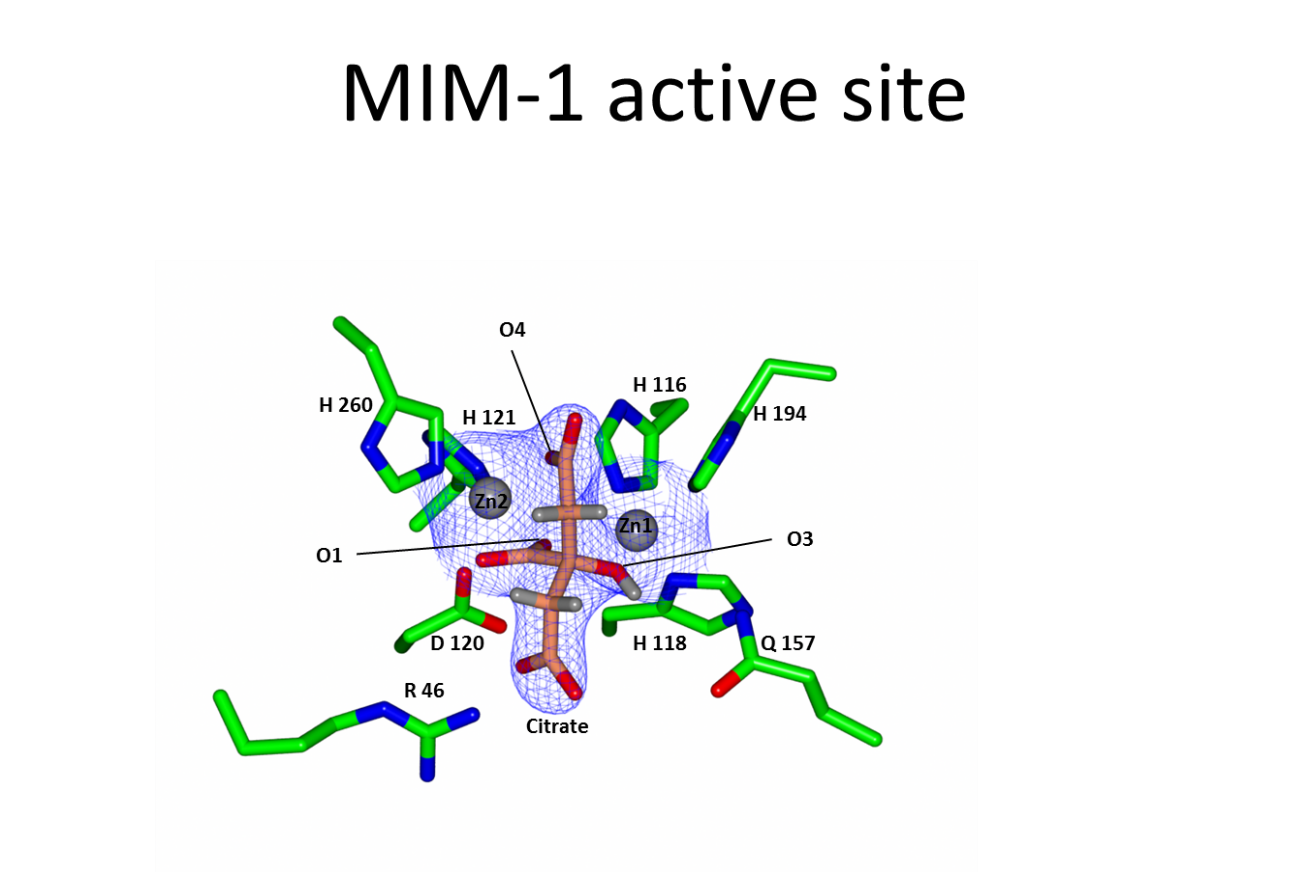

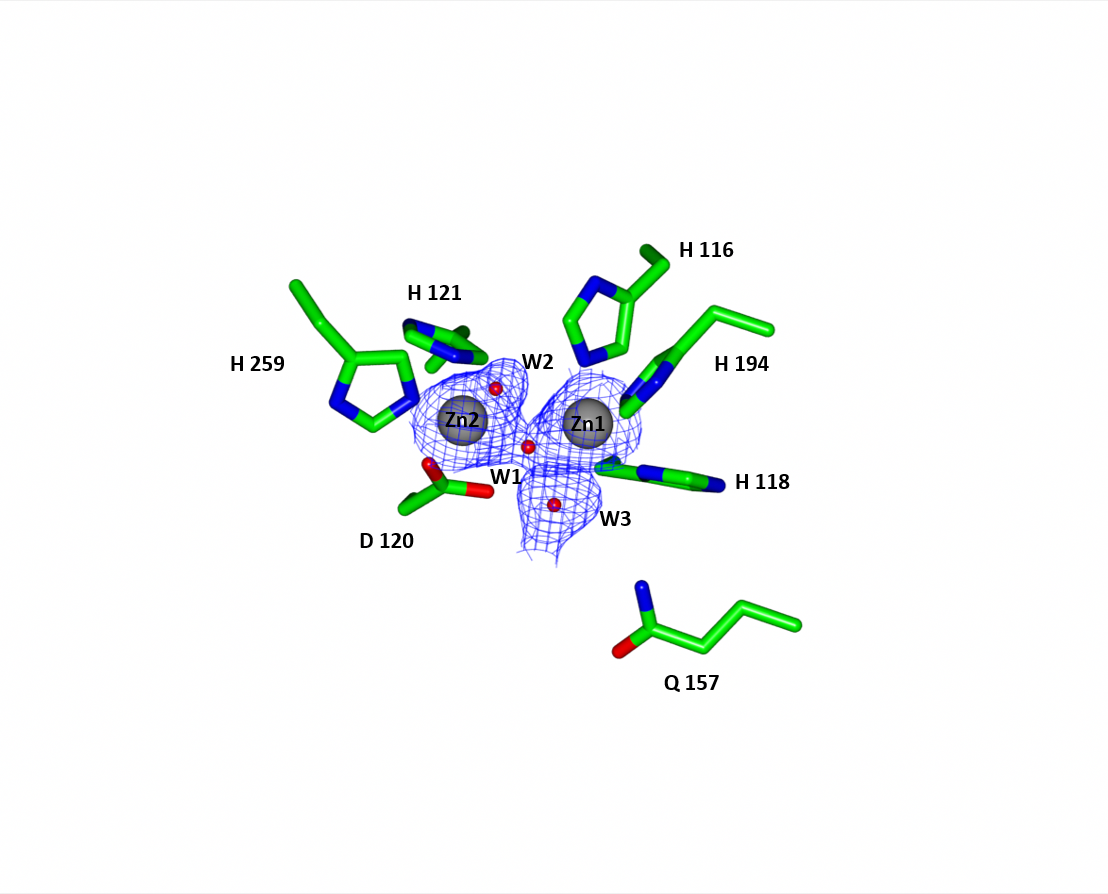


**Supplemental Figure 1.** Active site structures of MIM-1 (left, PDB 6AUF) and SAM-1 (right, PDB 6MFI), including Gln157 in both enzymes and Arg46 (for MIM-1 only), which may play an important role in substrate binding. The electronic density is displayed on the zinc metal ions and the citrate molecule (for MIM-1), and the water molecules W1, W2 and W3 (for SAM-1).

**Supplemental Table 1.** RMSD values for MIM-1 and SAM-1 against AIM-1 and L1 structures.

|  | RMSD values | | | |
| --- | --- | --- | --- | --- |
|  | AIM-1 | L1 | MIM-1 | SAM-1 |
| MIM-1  (276 Cα atoms) | 0.784  (218 Cα atoms) | 0.975  (198 Cα atoms) | - | 0.803  (217 Cα atoms) |
| SAM-1  (269 Cα atoms) | 0.601  (196 Cα atoms) | 1.08  (205 Cα atoms) | 0.803  (217 Cα atoms) | - |
